# Guideline adherence and patient satisfaction in the treatment of inflammatory bowel disorders – an evaluation study

**DOI:** 10.1186/1472-6963-9-17

**Published:** 2009-01-27

**Authors:** Claudia Pieper, Sebastian Haag, Stefan Gesenhues, Gerald Holtmann, Guido Gerken, Karl-Heinz Jöckel

**Affiliations:** 1 Institute for Medical Informatics, Biometry and Epidemiology, University Hospital of Essen, Essen, Germany; 2 Deptartment of Gastroenterology and Hepatology, University Hospital of Essen, Essen, Germany; 3 Institute for General Medicine, University Hospital of Essen, Essen, Germany; 4 Department of Gastroenterology, Hepatology and General Internal Medicine, Royal Adelaide Hospital, University of Adelaide, SA, Australia

## Abstract

**Background:**

Crohn's disease (CD) and ulcerative colitis (UC) are the most frequent inflammatory bowel disorders (IBD). IBD cause a significant burden to society due to extensive health care utilization from the first clinical symptoms until diagnosis and thereafter due to direct and indirect costs. Besides the socio-economic impact of CD and UC, gastrointestinal and extraintestinal symptoms affect quality of life, but there is remarkably little data about the quality of treatment as assessed by patient satisfaction, quality of life and adherence to guidelines. Thus the aim of this study was to identify variables that influence quality of treatment and quality of life as well as patient satisfaction.

**Methods:**

The Essener Zirkel Study was a cross sectional study of 86 IBD-patients with a confirmed diagnosis of CD or UC. They were recruited at primary, secondary and tertiary care settings. Quality of treatment, quality of life and patient satisfaction were evaluated. Consulting behaviour and number of examinations, duration of disease and variables regarding adherence to guidelines were evaluated, too.

**Results:**

59 (69%) patients had CD and 27 had UC (31%). 19% spent more than four years until the suspected diagnosis of IBD was confirmed and visited more than five physicians. All patients showed a significantly reduced quality of life compared to the 1998 German normative population. In spite of being under medical treatment, nearly half of the patients suffered from strong quality of life restricting symptoms. Over all, 35% described their treatment as moderate or bad. Patients who consulted psychotherapists and non-medical practitioners suffered significantly less from depression.

**Conclusion:**

Besides structural deficiencies due to the health care policy, we revealed the adherence to guidelines to be a problem area. Our findings support the assumption, that providing better health care and especially maintaining constant patient-physician communication improves patient satisfaction.

## Background

Crohn's disease (CD) and ulcerative colitis (UC) are the most frequent inflammatory bowel disorders (IBD) with a prevalence of approximately one in 500 [1]. In some instances, these diseases have overlapping clinical and pathological features, such as fever and submucosal edema [2]. A subgroup of patients was shown to have a slight increase in mortality [3], however, the primary aim of treatment is to induce and maintain remission as well as to prevent relapse. Main treatment options for managing IBD include enteral feeding, steroids to reduce inflammation, immunosuppressive agents and surgery in selected cases. IBD produces a range of gastrointestinal and extraintestinal symptoms, which greatly influence the patient's quality of life [4]. Despite significant therapeutic advances in recent years, patients require lifelong treatment in most cases. IBD is a chronic disease with early onset before 30 years of age and without medical cure. For these reasons, the burden of CD and UC is substantial on the health care system [5,6]. The chronic character of both CD and UC is characterized by periods of active disease and remission, and active periods of disease require long-lasting drug treatment and sometimes surgeries [7].

A wide net of hospitals and physicians is established in Germany. Medical facilities are appropriately equipped and the statutory health insurance system provides nearly full coverage for many medical treatments and medication. Independent of social status, nearly everybody in Germany has access to this system. Most people see a general practitioner at the first sign of health problems and they are free to see the general practitioner of their choice. Thus, general practitioners are often the first point of contact for patients. If necessary, the general practitioner refers patients to a specialist or a clinic. All hospitals in Germany, except for private clinics, offer services for insured patients.

The Essener Zirkel project aims to improve the quality of care at the interface between general practitioner, specialist and clinic. Focus groups discussed experiences and concerns and it became clear, that the patient's needs must be met. The quality of treatment has been increasingly recognized as an important aspect. It is particularly important in diseases such as UC and CD [8]. However, there is only limited information about it. Research has widely focussed on the pathophysiology and management of the diseases. Thus, the aim of the study was to identify factors that influence quality of care and patient satisfaction. The primary target variables were time between initial symptoms and diagnosis, information and coordination and symptom intensity. Furthermore, we evaluated guideline adherence for medical treatment as well as for number and type of examinations before and after diagnosis.

## Methods

The Essener Zirkel study was a cross sectional study in the city of Essen in the Ruhr area of Germany. It was realized within the framework of the Essener Zirkel project that aims to improve the quality of care of IBD. The city of Essen has approximately 585.000 inhabitants and ranges at sixth place among major German cities. Data was collected in 5 practices of general medicine, at 3 specialised gastroenterologists and in 3 tertiary care centres. We first identified hospitals specializing in IBD. All of them (N = 4) were then approached for recruitment. We assigned gastroenterologists (N = 15) and general practitioners (N = 34) who treat IBD-patients and divided them into two groups each according to the north-south divide in social class inequalities. Due to feasibility 50% of gastroenterologists and general practitioners (25% within each group) were then randomized using computer generated random numbers. Physicians were invited to participate in the study and to invite all presenting patients with a confirmed diagnosis of CD or UC during the recruiting period. Study participants gave written informed consent.

Patients had to be at between 18 and 75 years old to be included into this study. People with carcinoma, eating disorder, cardiovascular, endocrine or immunologic diseases and psychiatric diseases as well as pregnant women were excluded according to the study protocol.

Variables concerning quality of treatment and quality of life were evaluated using a standardized questionnaire. Clinical parameters were registered from all patients. Patient records were available if necessary. The Short Form Health Survey (SF-36) to assess Health Related Quality of Life (HRQOL) and the Hospital Anxiety and Depression Scale (HADS) to assess anxiety and depression were used [9-11]. Symptom intensity (SI) was assessed using a visual analogue scale (ranging from 1 to 9) [12]. Consulting behaviour and the number of examinations were evaluated using a questionnaire about consultation frequencies and examinations before and after diagnosing. The duration of the disease, clinical information, medication, concomitant diseases as well as sociodemographic information were obtained. The patient satisfaction with the professional level and attitude of the physician was assessed using a questionnaire that contained 10 items and was tested in a pilot study.

The ethical committee of the University of Duisburg-Essen approved the study. Proportions were expressed as percents. Values of the obtained clinical SI values were compared both between MC- and CU-patients with SPSS 12.0 (SPSS Inc., Chicago, IL, USA). To measure the degree of correlation between SI, HADS and the satisfaction scores the Pearson rank correlation test was used. All metric data are presented as means (± standard deviation). For comparison of groups the Wilcoxon rank test was used for continuous variables, the Fisher s exact test for dichotomous variables and the Chi-square test for ordinal variables. An alpha-error (p) of less than 5 per cent was considered significant.

## Results

### Patient characteristics

112 consecutive patients were potentially eligible for this study. 86 questionnaires returned from 5 general practitioners, 3 gastroenterologists and 3 clinics (response rate 77%). Among those 86 patients, 59 patients had CD (19 men, 40 women) and 27 had UC (8 men, 19 women). The mean age was 40.9 ± 12.1 years. A summary of sociodemographic characteristics is given in table 1.

**Table 1:** Patient characteristics

	**Ulcerative colitis**	**Crohn's disease**	**Total**
	(N = 27)	(N = 59)	(N = 86)
**Age **(mean value)	43.2 (10.5)	39.8 (12.7)	40.9 (12.1)

**Sex**	**N (%)**	**N (%)**	**N (%)**

Female	19 (70)	40 (68)	59 (69)

Male	8 (30)	19 (32)	27 (31)

26 patients could not be included in the study because of anxiety for loss of personal integrity, lack of interest or lack of time. 14 of these patients filled out a short questionnaire. 9 of them suffered from CD, 5 from UC (6 men, 8 women). The mean age was 41,7 ± 2,3 years.

### Time to diagnosis

Time to diagnosis means the duration of the diagnostic process, calculated from the first visit until the diagnosis was made. Table 2 gives an overview of the number of physicians visited and the duration until getting the diagnosis. 9% of the patients spent more than 2 years, 6% more than 3 years and 19% more than 4 years until getting the right diagnosis. Until then, 19% visited more than 5 physicians.

**Table 2:** Number of physicians visited until diagnoses and time until diagnosis

		**Ulcerative colitis**	**Crohn's disease**	**Total**
		(N = 27)	(N = 59)	(N = 86)
**Number of physicians visited until diagnosis**	1–2	55%	42%	49%
	
	3–4	22%	32%	27%
	
	5–6	7%	14%	11%
	
	more than 7	11%	5%	8%
	
	unknown	4%	5%	5%
	
	n/a		2%	1%

**Time until diagnosis**	same year	67%	38%	53%
	
	1 years	7%	17%	12%
	
	2 years	11%	7%	9%
	
	3 years	4%	7%	6%
	
	more than 4 years	12%	22%	17%
	
	unknown		7%	4%
	
	n/a		2%	1%

### Diagnostic procedures – Examinations during the last 12 month because of disease before diagnosing and before definite diagnosis

Before getting a confirmed diagnosis, 32 patients had no abdomen-ultrasound. 63% of the CD-patients and 25% of the UC-patients were examined by colonoscopy. 37% of the CD-patients and 25% of the UC-patients were examined by rectoscopy. Both groups did not significantly differ regarding radiological examinations (x-ray, computed-tomography (CT) or magnetic resonance imaging (MRI). A summary of all examinations is given in table 3.

**Table 3:** Examinations during the last 12 months because of disease and before definite diagnosis (absolute frequency)

**Examinations**	**Diagnosis**	**Total**	**Frequency (because of disease)**						**Frequency (before diagnosing)**					
			
			At no time	1 – 2 times	3 – 5 times	More than 6 times	Un-known	N/A	At no time	1 – 2 times	3 – 5 times	More than 6 times	Un-known	N/A
Abdominal ultrasound	CD	59	12	30	13	4			14	30	9	3	2	1
	
	UC	27	10	12	4			1	18	3	2	1	2	1

EGD	CD	59	35	18	3			3	29	20	6	1	3	
	
	UC	27	21	5				1	22	3			1	1

Colonoscopy	CD	59	19	31	8			1	21	30	6	1	1	
	
	UC	27	10	14	1	1		1	11	15				1

Rectoscopy	CD	59	35	19	1		2	2	28	17	2	3	6	3
	
	UC	27	17	4	3		1	2	14	12				1

X-ray of chest	CD	59	35	21		1		2	30	19	3		5	2
	
	UC	27	19	4	1		1	2	19	4	3			1

X-ray of stomach	CD	59	39	16	1		1	2	30	19	4	1	5	
	
	UC	27	22	3		1		1	19	6			1	1

CT of chest	CD	59	50	4			2	3	47	4			6	2
	
	UC	27	26					1	26					1

CT of stomach	CD	59	42	12	1		2	2	50	4	2		3	
	
	UC	27	22	4				1	25	1				1

MRI of chest	CD	59	55	1			1	2	55	1			2	1
	
	UC	27	24	1				2	16					1

MRI of stomach	CD	59	47	9			1	2	53	4			1	1
	
	UC	27	21	5				1	26					1

During one-year period (12 months) after being diagnosed with IBD, 13 patients had more than 3 radiological examinations. There was no significant difference between the groups regarding endoscopic examinations, abdomen-ultrasound or radiological examinations. 21 patients had more than 3 times abdomen-ultrasound. 10 patients had more than 3 colonoscopies during the last 12 months (table 3).

### Quality of life, Symptom Intensity

**Figure 1:**
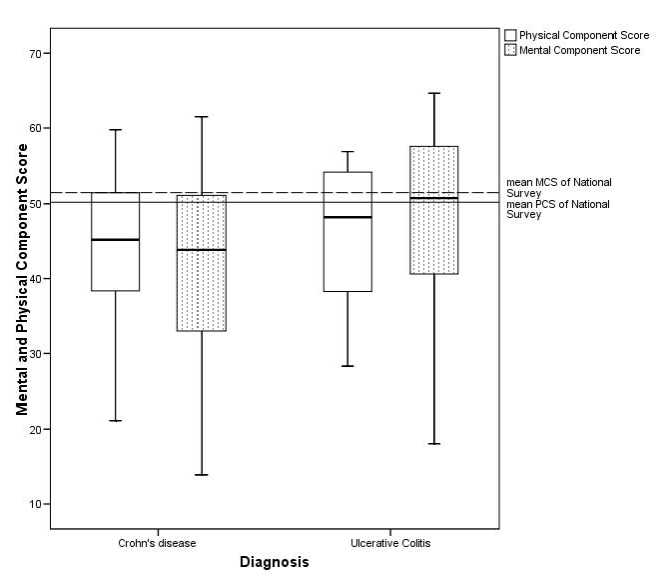
**Physical Component Summary (PCS) and Mental Component Summary (MCS)**. The German National Health Interview and Examination Survey included the SF-36-Questionnaire as an instrument for measuring health-related quality of life. A description of a German normative population sample is given as a result of the subjective assessment by 6964 survey participants aged between 18 and 80 years [10].

IBD patients had a significantly reduced quality of life (Physical Component Summary (PCS) and Mental Component Summary (MCS)) compared to the summary scales for the 1998 German normative population [10]: The overall values in our cohort were 45.1 ± 9.1 for PCS and 43.6 ± 12.0 for MCS. The overall values in the normative population were 49.1 for PCS and 50.2 for MCS. The difference between CD- and UC-patients is statistically significant (p = 0.01). The differences between CD-patients as well as UC-patients and the normative population are statistically significant, too (p = 0,000). Figure 1 shows the PCS and MCS values for both CD and UC. SI was significantly higher in tertiary care patients (5.3 ± 5.9) compared to patients in secondary (3.0 ± 3.5) and primary care (2.1 ± 2.2). 41 of the 86 patients at that time suffered from strong quality of life restricting symptoms and discomfort.

### Determinants of patient satisfaction

**Figure 2:**
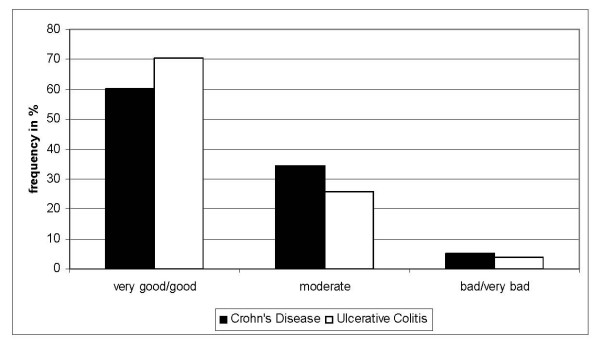
**Overall patient satisfaction**. 60% of the CD-patients and 70% of the UC-patients described their treatment as very good or good.

Over all, 60% of the CD-patients and 70% of the UC-patients described their treatment as very good or good. 40% of the CD-patients and 30% of the UC-patients described their treatment as moderate or bad. There differences between CD and UC were not statistically significant. Four most hampering problems were identified:

1. long duration until definite diagnosis (35%)

2. insufficient information for patients (26%)

3. an impersonally treatment (21%) and

4. a bad subjective coordination between general practitioner and gastroenterologist respectively general practitioner/gastroenterologist and clinic, which from the patient's point of view impinges negatively on the whole treatment (19%). More than a third of all patients (41%) consulted psychotherapists and non-medical practitioners. These patients gave significantly higher satisfaction ratings (p = 0.01). The time to diagnosis and the number of doctors visited were significantly correlated with patient satisfaction (p = 0.01): the longer the time to diagnosis the less satisfied the patients. Satisfaction ratings were not related to severity of disease activity. Figure 2 shows the overall patient satisfaction.

### Medication

Information on medication use was available in 84 of 86 cases. 5 patients (4%) were treated with a TNF-alpha-antibody. 29% received immunomodulators and 59% were treated with corticosteroids. 17% received 4 or more different medications, 21% received 3 different medications and 28% received 1 or 2 types of medication. Table 4 gives an overview of disease severity and medication category.

**Table 4:** Disease severity and medication category

	**Ulcerative colitis**	**Crohn's disease**	**Total**
	(N = 27)	(N = 58)	N=(84)
**Disease Severity**			

Mild	67%	49%	55%

Moderate	19%	29%	26%

Severe	4%	8%	7%

unknown	10%	14%	12%

**Medication category**			

Immunosuppressant	19%	34%	29%

Systemic steroids	26%	38%	32%

Topical steroids	22%	31%	27%

TNF-alpha-drug (any)		9%	4%

Mesalazine (derivative)	74%	41%	58%

## Discussion

The aim of the study was to evaluate the current situation of treatment and to identify variables that influence the quality of care and patient satisfaction.

In summary, the patients in our study had a significantly diminished quality of life compared to healthy controls as assessed by the 1998 German National Health Interview and Examination Survey [9]. A long duration for getting the definite diagnosis as well as insufficient information was ranked among the most urgent problems by the patients. In spite of being under medical treatment about half of the patients at that time suffered from quality of life restricting symptoms and discomfort. The number of patients who did not get abdominal-ultrasound before diagnosing is remarkable. UC-patients were less examined by colonoscopy resp. rectoscopy even though therapy guidelines suggest endoscopy intervals to provide confidence in the diagnosis [13].

Medical treatment for CD and UC has two main aims: to achieve or maintain remission. Therefore, treatment is aimed at controlling the permanent intestinal inflammation. In this population, as far as it could be ascertained, 29 (49%) CD-patients were in remission, 17 (29%) in moderate und 5 (8%) in severe active disease. Among the UC-patients, 18 (67%) had a mild, 5 (19%) a moderate and 1 patient had a severe active disease. Patients with extensive medication mainly were treated by specialists and in clinics. It is questionable whether

• too few patients were treated with immunomodulators

• the treatment with corticosteroids perhaps took too long

• a group of UC-patients was not treated with Mesalazine-derivate and if so, why.

However, a more detailed analysis of the medical therapy would exit the main focus of the study, but should be regarded in further investigations of this subject matter. Reddy et al already showed that IBD-patients did not get indicated medication and optimal doses but prolonged steroid therapy [8].

It has been shown that the subjective view of health is influenced not only by physical symptoms, but also by the level of worries and concerns about the disease [14]. The results of the consensus meeting by the Deutsche Gesellschaft für Verdauungs- und Stoffwechselkrankheiten e.V. (DGVS) describe new evidence-based data on psychosomatic treatment of patients with CD and UC [15]. But has it been put into action? We found that more than a third of all patients consulted psychotherapists and nonmedical practitioners. Satisfaction ratings of these patients were significantly higher. Moreover, patients who sought care from psychotherapists and non-medical practitioners suffered significantly less from depression. Randomized studies showed that psychotherapy could be superior to conventional medical therapy in improving outcomes such as symptoms intensity. Psychotherapy as an example is initially expensive but its benefits may increase over time and finally reduce consultations and health care costs [15]. However, patients often report that they have difficulty in getting information about the psychological consequences of their diseases. Many physicians are not able to devote as much time to the patient as they want. In consequence, a lot of patients seek care from psychotherapists and non-medical practitioners to get personalized care and to have access to information.

Combining additional treatment options with conventional treatment in a concept of integrated therapy could better meet patients need and improve outcomes [16]. Diseaserelated self-help organisations play an important role in the health care system in Germany. In our study population, IBD-patients who were members of a self-help group had an increased knowledge about the disease.

However, the results of our study must be interpreted with some caution, since the limitations of this study have to be taken into account. For instance the sample is no average sample, among others due to the design of the study and therefore does not enable us to generalize e.g. the correlation of time to diagnosis and satisfaction ratings. In contrast to the planned number of cases, a smaller number of patients was involved in the study. While it was intended to include 200 patients with IBD, data from 86 of 112 patients became available. The short recruitment period turned out to be difficult in high-frequented gastroenterology practices. It was not possible to prolong the survey itself because of the schedule of the overall project.

But the number of recruited patients is very similar to the rates observed in other trials in this field [17,18]. Even though the sample size may limit generalisation of our data, we assessed all patients who presented at chosen practices and hospitals during the study period and we achieved a response rate of 77%. Assessing data from non-participants to analyse for potential non-responder bias, we did not observe a statistically significant difference between patient characteristics. It has to be emphasized, that primary and secondary care patients as well as tertiary care patients were involved in this study. Because we did not only recruit patients in a tertiary care centre, there was no single sided preselection and patients in different stages of the disease were included. Because of the cross sectional design, a firm conclusion about the causal relation between SI and consultation behaviour cannot be drawn, but we found at least a statistical association between quality of care and the investigated outcomes, that may have public health relevance.

In Germany guidelines for IBD have existed for several years. We revealed the adherence to guidelines to be a problem area in the treatment process. This finally affects the quality of care concerning the physical condition as well as patient satisfaction and quality of life.

Moreover, a regular problem is the time for physician-patient-communication and information. Structural deficiencies, due to the health care policy, such as finance, insurance and politics have a great impact on the time physicians spend with patients [19].

## Conclusion

Our findings support the assumption that maintaining constant physician-patient-communication improves patient satisfaction. Cooperation between primary, secondary and tertiary care was found to have an impact on patient satisfaction, too. Despite of a well-established cooperation in the local gastroenterological field, our results showed improvement options in the treatment of IBD-patients. In the city of Essen, improvement options concerning the problems mentioned before have been discussed jointly. Concrete actions have been developed and implemented in cooperation with the enlisted practices and clinics as well as representatives of patient organisations. Therefore the Essener Zirkel project has become member of the initiative "Gesundes Land Nordrhein-Westfalen" of the Department of Health of North Rhine-Westphalia.

Research in this area is fragmented, further investigations should address the problem of involving larger proportions of IBD patients in primary, secondary and tertiary care.

## Competing interests

The authors declare that they have no competing interests.

## Authors' contributions

CP carried out the study, participated in its design and coordination, performed the statistical analysis and drafted the manuscript. SH carried out the study, participated in its design and drafted the manuscript. SG provided support for the study. GH participated in the design of the study. GG provided support for the study. KHJ participated in the design of the study and its coordination. All authors have seen and approved the final manuscript.

## Pre-publication history

The pre-publication history for this paper can be accessed here:



## References

[B1] Shivanada S, Lennard-Jones J, Logan R, Fear N, Price A, Carpenter L, van Blankenstein M, the EC-IBD Study Group (1996). Incidence of inflammatory bowel disease across Europe: is there a difference between north and south? Results of the European collaborative study on inflammatory bowel disease (EC-IBD). Gut.

[B2] Kirsner JB, Shorter R (1994). Diseases of the Colon, Rectum, & Anal Canal.

[B3] Jess T, Loftus EV, Harmsen WS, Zinsmeister AR, Tremaine WJ, Melton J, Munkholm P, Sandborn WJ (2006). Survival and cause-specific mortality in patients with inflammatory bowel disease: a long-term outcome study in Olmsted County, Minnesota, 1940–2004. Gut.

[B4] Maunder RG, de Rooy EC, Toner BB, Greenberg GR, Steinhart AH, McLeod RS, Cohen Z (1997). Health-related concerns of people who receive psychological support for inflammatory bowel disease. Can J Gastroenterol.

[B5] Beiche A, Konig HH, Ebinger M, Matysiak-Klose D, Braun V, Leidl R (2003). Costs of ambulant care for patients with inflammatory bowel disease in general. Z Gastroenterol.

[B6] Bassi A, Dodd S, Williamson PR, Bodger K (2004). Cost of illness of inflammatory bowel disease in the UK: a single centre retrospective study. Gut.

[B7] Hanauer SB, Present DH (2003). The state of the art in the management of inflammatory bowel disease. Rev Gastroenterol Disord.

[B8] Reddy SI, Friedman S, Telford JJ, Strate L, Ookubo R, Banks PA (2005). Are Patients with Irritable Bowel Disease receiving optimal care. Am J Gastroenterol.

[B9] Bellach BM, Knopf H, Thefeld W (1998). Der Bundes-Gesundheitssurvey 1997/98. Das Gesundheitswesen. Sonderheft (Stuttgart Thieme).

[B10] Bullinger M, Kirchberger I (1998). SF-36 Fragebogen zum Gesundheitszustand. Handanweisung.

[B11] Hinz A, Schwarz R (2001). Anxiety and depression in the general population: Normal values in the Hospital Anxiety and Depression Scale. Psychother Psychosom Med Psychol.

[B12] Nyrén O, Adami HO, Bates S, Bergström R, Gustavsson S, Lööf L, Sjödén PO (1987). Self-rating of pain in nonulcer dyspepsia. A methodological study comparing a new fixed-point scale and the visual analogue scale. J Clin Gastroenterol.

[B13] Hoffmann JC, Zeitz M, Bischoff SC, Brambs HJ, Bruch HP, Buhr HJ, Dignass A, Fischer I, Fleig W, Folsch UR, Herrlinger K, Hohne W, Jantschek G, Kaltz B, Keller KM, Knebel U, Kroesen AJ, Kruis W, Matthes H, Moser G, Mundt S, Pox C, Reinshagen M, Reissmann A, Riemann J, Rogler G, Schmiegel W, Scholmerich J, Schreiber S, Schwandner O, Selbmann HK, Stange EF, Utzig M, Wittekind C (2004). Ergebnisse einer evidenzbasierten Konsensuskonferenz der Deutschen Gesellschaft für Verdauungs- und Stoffwechselkrankheiten zusammen mit dem Kompetenznetz chronisch entzündliche Darmerkrankungen. Z Gastroenterol.

[B14] Drossmann DA, Kirsner JB (2000). Psychosocial factors in ulcerative colitis and crohn s disease. Inflammatory Bowel Disease.

[B15] Moser G, Leitlinien der DGVS (2003). Morbus Crohn, Psychosomatik. Konsensus der Deutschen Gesellschaft und Verdauungs- und Stoffwechselkrankheiten. Z Gastroenterol.

[B16] Elsenbruch S, Langhorst J, Popkirowa K, Müller T, Luedtke R, Franken U, Paul A, Spahn G, Michalsen A, Janssen OE, Schedlowski M, Dobos GJ (2005). Effects of mind-body therapy on quality of life and neuroendocrine and cellular immune functions in patients with ulcerative colitis. Psychotherapy & Psychosomatic.

[B17] Baumgart DC, Pintoffl JP, Sturm A, Wiedenmann B, Dignass AU (2006). Tacrolimus is safe and effective in patients with severe steroid-refractory or steroid-dependent inflammatory bowel disease-a long-term follow-up. Am J Gastroenterol.

[B18] Bokemeyer B (2004). Klinische Studien in Deutschland. Der niedergelassene Gastroenterologe als Partner in klinischen Studien. Dtsch med Wochenschr.

[B19] Magee M, Hojat M (2001). Impact of health care system on physicians' discontent. J Community Health.

